# Early Manifestations of Toxic Epidermal Necrolysis

**DOI:** 10.5811/cpcem.2017.6.34371

**Published:** 2017-10-03

**Authors:** James Chapman, Katy Deblois

**Affiliations:** Kent Hospital, Department of Emergency Medicine, Warwick, Rhode Island

## CASE PRESENTATION

A 26-year-old female presented to the emergency department (ED) with complaint of vaginal irritation 11 days after starting trimethoprim/sulfamethoxazole (TMP/SMX) for a paronychia. She was initially treated as an outpatient with cephalexin and TMP/SMX without improvement. On day nine of TMP/SMX, the paronychia was drained.

**Image 1:**
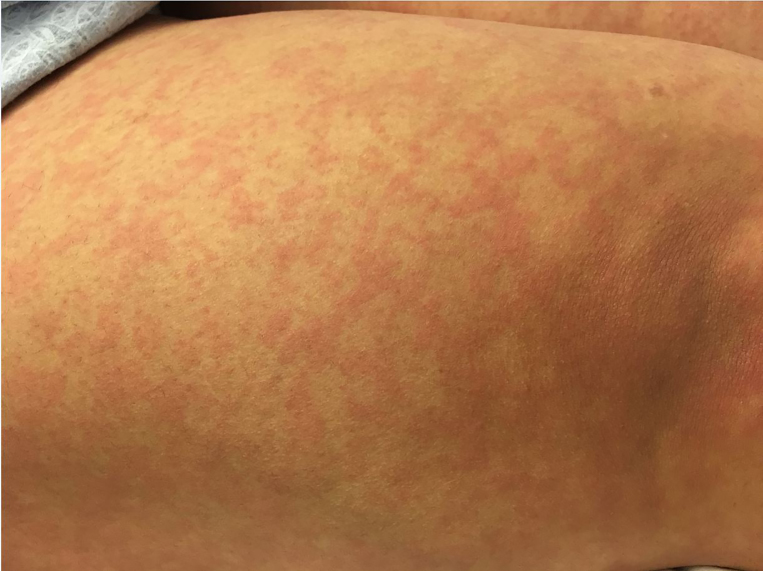
Initial rash on leg in toxic epidermal necrolysis (Day 1)

**Image 2:**
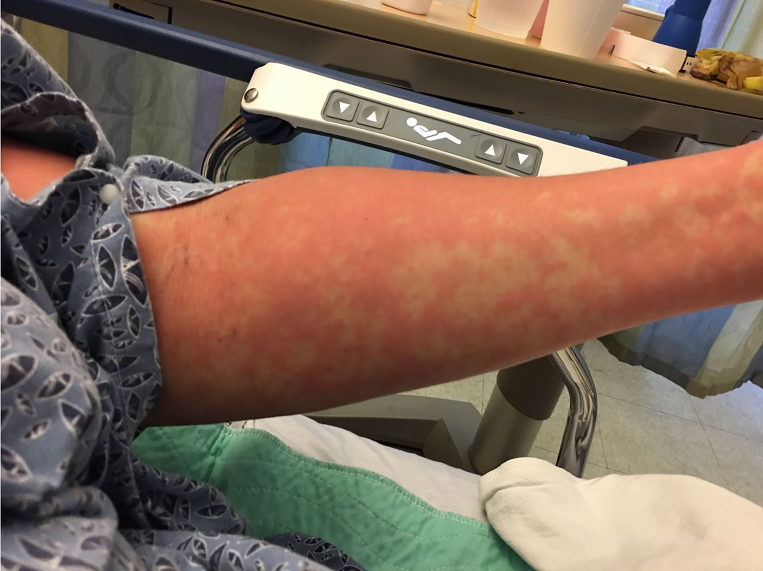
Progressing rash in toxic epidermal necrolysis, approximately eight hours after initial presentation.

**Image 3:**
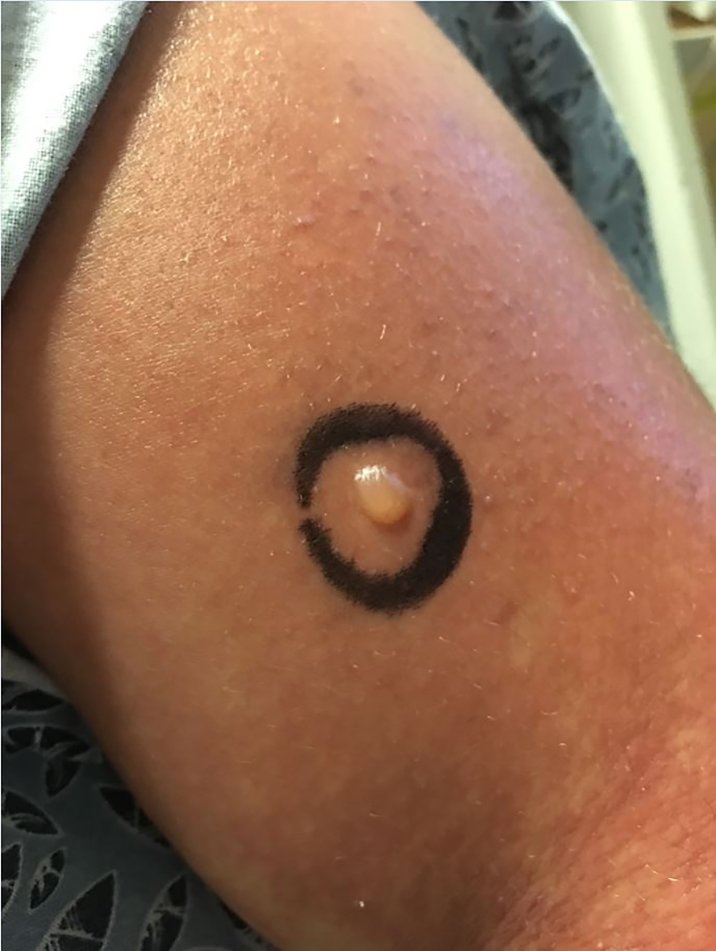
Initial bulla (circle) on upper extremity in toxic epidermal necrolysis, approximately 22 hours after initial presentation.

Physical examination included normal vitals and normal pelvic findings, but did reveal an erythematous macular non-pruritic rash beginning at her chest (Image 1). Complete blood count and basic metabolic panel were unremarkable. The combination of her skin findings with recent TMP/SMX administration led to a diagnosis of probable early Stevens-Johnson syndrome (SJS), or toxic epidermal necrolysis (TEN). The patient was transferred to a burn center where symptoms worsened (Image 2 and 3). She had sloughing of her vaginal and oral mucosa, and skin on her face, trunk, and extremities. In total, over 40% of her total body surface area was affected. She was discharged home after 16 days with a diagnosis of TEN.

## DISCUSSION

SJS/TEN is a life-threatening condition of the skin and mucous membranes due to immune-complex-mediated hypersensitivity.^1^ Medications, including sulfonamides such as TMP/SMX, are frequently linked to SJS.^2^,^3^ Over the last decade with the increased incidence of community-associated methicillin-resistant Staphylococcus aureus, sulfonamides are increasingly prescribed. The disease initially manifests as a flu-like illness and progresses to a macular non-pruritic rash that begins on the trunk. This rash progresses to bullae and necrosis of the entire layer of the dermis. Even with early diagnosis and management, mortality ranges from 20–30%.^4^ It is imperative that physicians consider SJS as a diagnosis when a patient presents with new skin findings. Additionally, this case reminds physicians of the severe and possibly deadly side effects of commonly used medications.^5^ Finally, physicians should use caution in providing prescriptions, especially when another intervention such as an incision and drainage would provide more appropriate care.

**CPC-EM Capsule**

What do we already know about this clinical entity?

We know that the disease processes seen in Stevens Johnson Syndome and Toxic Epidermal Necrolysis are one of the few potentially deadly dermatologic emergencies that must be screened for in the emergency department. We also know that there are many causes of this disease process including medications, infections, and genetics.

What is the major impact of the image(s)?

The images in this case report show the early manifestations of SJS/TEN. When looking through the literature, most case reports do not have the early images. It is important for physicians to recognize the early manifestations of this disease process so patients are not sent home early, and can be appropriately treated.

How might this improve emergency medicine practice?

This will help physicians recognize this potentially fatal disease early, and begin treatment earlier.
